# Whole sporozoite immunization with *Plasmodium falciparum* strain NF135 in a randomized trial

**DOI:** 10.1186/s12916-023-02788-9

**Published:** 2023-04-07

**Authors:** Saskia C. van der Boor, Manon Alkema, Geert-Jan van Gemert, Karina Teelen, Marga van de Vegte-Bolmer, Jona Walk, Reinout van Crevel, Quirijn de Mast, Christian F. Ockenhouse, Robert W. Sauerwein, Matthew B. B. McCall

**Affiliations:** 1 Department of Medical Microbiology, Radboud Center for Infectious Diseases, Radboud University Medical Center, Geert Grooteplein Zuid 28, 6525 GA Nijmegen, The Netherlands; 2 Present affiliation: TropIQ Health Sciences, Transistorweg 5-C02, 6534 AT Nijmegen, The Netherlands; 3 Department of Internal Medicine, Radboud Center for Infectious Diseases, Radboud University Medical Centre, Geert Grooteplein Zuid 10, 6525 GA Nijmegen, The Netherlands; 4 PATH Malaria Vaccine Initiative, Washington, DC 20001 USA

**Keywords:** Malaria, *Plasmodium falciparum*, Whole sporozoite immunization, Vaccine-induced protection, NF135

## Abstract

**Background:**

Whole sporozoite immunization under chemoprophylaxis (CPS regime) induces long-lasting sterile homologous protection in the controlled human malaria infection model using *Plasmodium falciparum* strain NF54. The relative proficiency of liver-stage parasite development may be an important factor determining immunization efficacy. Previous studies show that *Plasmodium falciparum* strain NF135 produces relatively high numbers of large liver-stage schizonts in vitro. Here, we evaluate this strain for use in CPS immunization regimes.

**Methods:**

In a partially randomized, open-label study conducted at the Radboudumc, Nijmegen, the Netherlands, healthy, malaria-naïve adults were immunized by three rounds of fifteen or five NF135-infected mosquito bites under mefloquine prophylaxis (cohort A) or fifteen NF135-infected mosquito bites and presumptive treatment with artemether/lumefantrine (cohort B). Cohort A participants were exposed to a homologous challenge 19 weeks after immunization. The primary objective of the study was to evaluate the safety and tolerability of CPS immunizations with NF135.

**Results:**

Relatively high liver-to-blood inocula were observed during immunization with NF135 in both cohorts. Eighteen of 30 (60%) high-dose participants and 3/10 (30%) low-dose participants experienced grade 3 adverse events 7 to 21 days following their first immunization. All cohort A participants and two participants in cohort B developed breakthrough blood-stage malaria infections during immunizations requiring rescue treatment. The resulting compromised immunizations induced modest sterile protection against homologous challenge in cohort A (5/17; 29%).

**Conclusions:**

These CPS regimes using NF135 were relatively poorly tolerated and frequently required rescue treatment, thereby compromising immunization efficiency and protective efficacy. Consequently, the full potential of NF135 sporozoites for induction of immune protection remains inconclusive. Nonetheless, the high liver-stage burden achieved by this strain highlights it as an interesting potential candidate for novel whole sporozoite immunization approaches.

**Trial registration:**

The trial was registered at ClinicalTrials.gov under identifier NCT03813108.

**Supplementary Information:**

The online version contains supplementary material available at 10.1186/s12916-023-02788-9.

## Background

The resurging trend in clinical cases and deaths caused by malaria infection highlights the need for a durable and effective vaccine, particularly against *Plasmodium falciparum* (*Pf*), the species responsible for the greatest burden of morbidity and mortality [1].

Whole sporozoite vaccines, by virtue of exposing the host immune system to the full repertoire of pre-erythrocytic parasite antigens, have the advantage of inducing a broader, more robust immune response than sub-unit vaccines can. Immunization with live *Pf* sporozoites under cover of chemoprophylaxis (CPS regime) represents the most efficient known approach to inducing sterile protection in malaria-naïve individuals. CPS is generally conducted using a blood schizonticide drug such as chloroquine or mefloquine. These allow full liver-stage development of the immunizing sporozoite inoculum and subsequent release of a first generation of blood-stage parasites from the liver into circulation, where they succumb to the chemoprophylaxis during their first round of intraerythrocytic schizogony. Exposure to the bites of as few as forty-five mosquitoes infected with the NF54 strain of *Pf* under chloroquine prophylaxis, spread across three rounds of immunization, is sufficient to induce sterile homologous protection in 100% of study participants [2, 3]. Direct intravenous administration of GMP-produced, irradiated, aseptic, purified, cryopreserved, infectious NF54 *Pf* sporozoites under chloroquine chemoprophylaxis (*Pf*SPZ-CVac) similarly results in high-level homologous as well as heterologous protection in malaria-naïve individuals [4–6]. *Pf*SPZ-CVac also induced homologous protection in life-long malaria-exposed Equatoguinean adults, albeit at slightly lower levels (~ 55%), and a phase 2 trial is underway in Mali [7].

Other methods of whole sporozoite immunization include sub-lethal irradiation and genetic attenuation of sporozoites [8, 9]. However, relatively higher sporozoite inocula are required than for CPS [4, 5, 8, 10]. It is thought that this is because irradiated and first-generation genetically attenuated sporozoites terminally arrest early during liver-stage development, limiting the biomass and breadth of antigen expression compared to that in late liver-stage schizonts.

Notably, all these attenuated sporozoite vaccine approaches have used the same laboratory *Pf* strain NF54 (originally of West African origin). We have previously shown that there is an intrinsic difference between parasite strains in their ability to infect and multiply within human hepatocytes [11, 12]. In vitro, the Cambodian *Pf* strain NF135 infects fresh primary human hepatocytes at two- to fourfold higher rates compared to NF54, and NF135 liver-stage schizonts are bigger and contain greater numbers of merozoites [11, 12]. Moreover, in the controlled human malaria infection (CHMI) model, infection with five NF135-infected mosquito bites results in a liver-to-blood inoculum that is approximately tenfold higher compared to a historic control with the same number of NF54-infected mosquito bites without significantly increasing the number or severity of adverse events [11, 13]. Given the dose-dependency of whole sporozoite immunization inocula, the greater liver-stage biomass induced by NF135 could be of benefit in generating protective immune responses, but this has not yet been evaluated. The primary objective of the current proof-of-principle study was to assess the safety and tolerability of NF135 immunizations using the CPS approach.

## Methods

### Study design and participants

This open-label, single-center trial was conducted at the Radboud University Medical Center (Radboudumc) in Nijmegen, the Netherlands, between April 2019 and February 2021. The study population was comprised of healthy, malaria-naïve, adults aged 18–35 years old at time of first immunization. Upon informed consent, participants were screened for medical and family history, physical examination, complete blood counts, clinical chemistry, and serologic analysis for human immunodeficiency virus, active hepatitis B and C, and asexual *Pf* parasites. Additionally, participants in cohort A were screened for contra-indications to mefloquine prophylaxis. All participants received a urine toxicology test and female participants were additionally tested for pregnancy.

### Study procedures

**Fig. 1:**
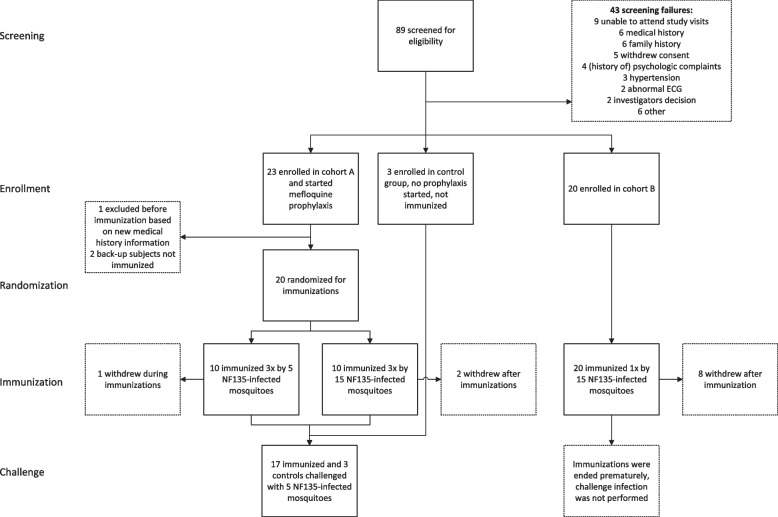
Flow chart. Eighty-nine candidates were tested for eligibility and fourty-six were included in the study after screening: twenty-three in cohort A (three additional participants started mefloquine prophylaxis in case of dropouts prior to the first immunization), twenty in cohort B, and three in the control groups. Participants in cohort A were randomized to receive immunizations with either three times fifteen NF135-infected mosquitoes (high-dose) or three times five NF135-infected mosquitoes (low-dose). One participant dropped out after the first immunization and two participants after the third immunization, all three because of personal or logistical reasons. Participants in cohort B received one immunization with fifteen NF135-infected mosquitoes (high-dose). The control group was not immunized. Immunized participants from cohort A, and three malaria-naïve control participants were challenged by bites of five NF135-infected mosquitoes. In cohort B, the trial was prematurely ended after the first immunization and participants were not subsequently challenged

Cohort A participants (*n* = 20) were included on 1 April 2019 and started weekly treatment with 250 mg mefloquine prophylaxis three weeks prior to the first CPS immunization. The NF135 strain has reduced sensitivity to chloroquine but similar sensitivity to mefloquine as NF54 in vitro [14]. Three additional participants started mefloquine prophylaxis in case of dropouts prior to the first immunization. As a safety precaution, 10 days after starting mefloquine prophylaxis, participants were asked to complete the Depression Anxiety Stress Scales (DASS) questionnaire and twenty items on the Community Assessment of Psychic Experiences (CAPE) questionnaire to detect any mefloquine-induced changes in emotional states including depression, anxiety, stress, and psychotic symptoms. Twenty participants in cohort A were subsequently randomized to receive three immunizations, spaced one month apart, by the bites of either fifteen (*n* = 10, high-dose) or five (*n* = 10, low-dose) *Anopheles stephensi *(*An. stephensi*) mosquitoes infected with the C10 clone of the *Pf* NF135 strain (NF135.C10) (Fig. 1). After each immunization, mosquitoes were examined to verify that they had taken a blood meal and to confirm the presence of sporozoites in their salivary glands. Participants underwent immunization with additional mosquitoes if insufficient infected mosquitoes had taken a blood meal.

A safety monitoring committee was appointed to evaluate safety data at prespecified time points throughout the trial: after each immunization, at day 21 and 35 after CHMI, and *ad ho*c upon the occurrence of any predefined study holding criteria.

Follow-up took place from day 6 until day 10 after each immunization in an outpatient setting. Blood samples were collected daily for safety follow-up and retrospective qPCR assessment. Additionally, a thick smear was performed on days 7, 8, and 9 after immunization or if participants were symptomatic. Symptomatic participants with a positive thick smear were treated with a standard curative regimen of 1000 mg atovaquone and 400 mg proguanil once daily for 3 days.

Nineteen weeks after the third immunization, participants underwent a CHMI with five NF135-infected mosquitoes using similar procedures as for immunization. After challenge infection, participants were followed up once daily for safety lab measurements and prospective qPCR from day 6 until day 21 post-challenge. All participants were treated with a curative regimen of atovaquone/proguanil at the time of detection of parasitemia or ultimately 28 days after challenge infection.

Cohort B participants were included on 31 August 2021, following a COVID-19 pandemic-related delay. Cohort B participants (*n* = 20) were scheduled to all receive three immunizations by the bites of fifteen *Pf* NF135-infected *An. stephensi* mosquitoes (high-dose) and were originally intended to receive mefloquine prophylaxis (and if necessary, atovaquone/proguanil rescue treatment) as in cohort A. However, due to unanticipated ongoing parasite multiplication under mefloquine prophylaxis in cohort A, and the longer observed atovaquone half-life than is reported in the summary of product characteristics [15], the study protocol was subsequently amended and received ethics approval for cohort B, as follows: instead of chemoprophylaxis, cohort B participants received standard presumptive treatment with 80 mg artemether and 480 mg lumefantrine twice daily over 3 days, starting on day 7 after immunization. Participants were followed up from day 6 up to day 10 after immunization as outpatients, and a prospective qPCR was performed daily to monitor treatment efficacy. Under the amended protocol, cohort B participants were moreover scheduled to be randomized to undergo either homologous (NF135, *n* = 10) or heterologous (NF54, *n* = 10) CHMI 19 weeks after their third immunization. However, cohort B was ultimately prematurely terminated after the first immunization due to withdrawal of eight participants following a change in the trial schedule as a result of (i) the COVID-19 pandemic and (ii) the occurrence of a serious adverse event.

### Study outcome measures

To determine the safety and tolerability of NF135 sporozoite immunization under chemoprophylaxis, the prespecified primary endpoint was the frequency and the severity of adverse events after NF135 CPS immunization. To determine the dose-dependent protective efficacy of NF135 CPS immunizations, secondary endpoints included the number of sterilely protected participants and the time to parasitemia detectable by qPCR after homologous and heterologous NF54 challenge infection. Original secondary objectives additionally included assessment of the protective efficacy against a second heterologous strain NF166 and assessment of protection against homologous or heterologous re-challenge 1 year after immunizations in those participants protected against respectively homologous or heterologous CHMI at 19 weeks. Following the compromised immunizations in cohort A, the study protocol for cohort B was amended and received ethics approval to prioritize comparison of homologous (NF135) versus heterologous (NF54) efficacy over protection against two different heterologous strains and to perform re-challenge only if protection at 19 weeks was ≥ 50%. Ancillary endpoints included the peak parasite density and parasite density on day 7 after each immunization.

### Study approval

The study protocol (file number NL63594.091.17) and its amendments were approved by the Netherlands’ Central Committee on Research Involving Human Subjects, and the Western Institutional Review Board, and the trial is registered at ClinicalTrials.gov under identifier NCT03813108. The full clinical trial protocol can be accessed at ClinicalTrials.gov.

### Parasite culture and generation of infectious mosquitoes

The *Pf* NF135 strain originates from a clinical isolate from Cambodia, and the NF135.C10 clone was obtained by limiting dilution and characterized for use in CHMI [14]. Parasites were cultured in a semi-automated culture system, as described previously [16]. *An. stephensi* mosquitoes were reared at the insectary of the Radboudumc and infected by feeding on gametocyte cultures through standard membrane feeding [16].

### Quantification of parasites by qPCR

Parasite densities were quantified daily and retrospectively (cohort A) or prospectively (cohort B) on days 6 to 10 after immunization by real-time quantitative PCR (qPCR) and prospectively on days 6 to 21 after challenge (cohort A). qPCR was performed on the 18S ribosomal RNA genes as described previously [17]. The prepatent period was determined by the time to parasitemia, defined by the time to the first qPCR measurement with a parasite density greater than 100 parasites per mL of blood.

### Randomization

Participants of cohort A were randomly allocated to the high- or low-dose immunization groups. A study member not directly involved in participant follow-up was responsible for creating a computer-generated list with random numbers to which the participants were assigned. A second study member was responsible for checking whether randomization had occurred correctly.

### Safety assessment

All participants received a memory aid booklet to register symptoms and an oral thermometer to record daily temperature. Adverse events were recorded by the attending physician and graded as mild (easily tolerated, grade 1), moderate (interfering with daily activity, grade 2), or severe (preventing daily activity, grade 3) and in the case of fever as mild (38.0–38.4 °C), moderate (38.5–38.9 °C), or severe (≥ 39 °C). Adverse events were categorized by the International Classification of Diseases 10 code. Systemic solicited adverse events were defined as: fever (by examination), headache, fatigue, malaise, chills, myalgia, dizziness, sweats, nausea, vomiting, abdominal pain, diarrhea, and chest pain. Local solicited adverse events were tenderness, induration, erythema, swelling, pain, and pruritis. Causality to the study procedures was categorized as not related, possibly related, or probably related. Safety blood tests were performed once daily at each study visit, including hematology and biochemistry safety evaluations (including LDH and highly sensitive troponin T) and liver function tests. A thick smear was performed on days 7, 8, and 9 after immunization (cohort A) or if participants were symptomatic (cohort A and B) to detect breakthrough infection. 0.5 μL of blood was assessed by microscopy and considered positive if two unambiguous parasites were detected. Mefloquine and atovaquone levels were measured retrospectively in citrate-plasma by liquid chromatography (limit of detection 20 ng/mL). Artemether and lumefantrine levels were measured retrospectively in citrate-plasma by high performance liquid chromatography coupled to tandem mass spectrometry [18].

### In vitro drug susceptibility assays

Circulating breakthrough parasites were re-cultured and susceptibility to mefloquine in serial dilutions was determined at 0.83% parasitemia and 3% hematocrit. After incubation for 72 h at 37 °C, parasites were lysed and relative parasitemia was determined by measuring DNA bound SYBR green fluorescent signal.

### Measurement of anti-sporozoite antibody titers

Nunc MaxiSorp™ 96-wells plates (ThermoFisher) were coated overnight at 4 °C with 100 μl, equivalent to the lysate of 40,000 *Pf* sporozoites, per well. Plates were blocked with 5% skimmed milk in PBS and subsequently incubated with a 1:100 dilution of sera. Detection was done with 1:40,000 dilution Goat anti-Human IgG HRP (Invitrogen, Cat. No. 31412). ELISAs were developed by adding 100 μL tetramethylbenzidine and stopped with 50 μL 0.2 M H2SO4. Absorbances were read at 450 nm on an iMark™ microplate absorbance reader (Bio-Rad).

ELISA analyses were performed using Auditable Data Analysis and Management System for ELISA (ADAMSEL FPL v1.1).

A pool of 100 sera from adults living in an area in Tanzania where malaria is highly endemic served as positive control serum. The standard curve was plotted on a logarithmic scale and fitted to a power trend line (R2 > 0.99). Optical density (OD) measurements for each test sample were converted to arbitrary units (AU) relative to the control serum (control serum was set at 100) and normalized for the Xmid of each plate.

### Statistical analysis

Based on previous CPS immunization studies and by convention, twenty participants were recruited per cohort. This sample size allowed for a delay in time to parasitemia of 0.5 days after homologous challenge (*α* = 0.05 and *β* = 0.80). Statistical analyses were performed using the GraphPad Prism software (version 9, GraphPad Software Inc., California, USA). Fisher’s exact test was used for categorical comparison between study groups. For comparison of continuous variables between study groups, the Mann–Whitney *U* test was used. Friedman test with *post hoc* paired comparison using Bonferroni-corrected Wilcoxon signed rank test was used for comparisons of time points within studies.

### Role of the funding source

This study was funded by PATH’s Malaria Vaccine Initiative. The financial sponsor was involved in the design of the study and the analysis and interpretation of the results and contributed to writing and reviewing this report.

## Results

### Recruitment and retention

From 2 February 2019 to 28 August 2020, forty-six malaria-naïve adults were enrolled (Fig. 1). Baseline characteristics were comparable between groups (Table 1). The median age was 24 (range 19–30) at time of enrolment and 53% of participants were female. In cohort A, twenty-three participants commenced with mefloquine prophylaxis 3 weeks prior to the first immunization. One reserve participant was excluded from enrolment due to mefloquine-related insomnia. In total, twenty participants commenced CPS immunizations. In the low-dose immunization group of cohort A, one participant withdrew from the study after the first immunization and two participants withdrew after their third immunization in the high-dose group, all for personal or logistical reasons. Participants in cohort B did not receive mefloquine prophylaxis. Of twenty participants who underwent a first immunization followed by presumptive artemether/lumefantrine treatment, one participant was subsequently withdrawn due to a serious adverse event. Seven other participants withdrew after the first immunization following changes in the trial schedule as a result of the serious adverse event and the COVID-19 pandemic. It was considered insufficiently justified to continue with the remaining number of participants, and the trial was therefore prematurely ended after one immunization.

**Table 1:** Baseline characteristics

		High dose	Low dose	Controls	All
		*n* = 30	*n* = 10	*n* = 3	*n* = 43
Immunization dose (# infectious mosquitoes per immunization)		15	5	NA	NA
Sex, # female (%)		17 (57)	4 (40)	2 (67)	23 (53)
Age (years)		24 (19–30)	24.5 (21–29)	21 (20–24)	24 (19–30)
Body mass index (kg/m^2^)		23.8 (18.1–29.9)	22.8 (19.4–29.7)	22.0 (21.9–23.2)	23.2 (18.1–29.9)
Race (%)					
	Caucasian	23 (77)	8 (80)	3 (100)	34 (79)
	Asian	4 (13)	2 (20)	NA	6 (14)
	Unknown	1 (3)	NA	NA	1 (2)
	More than one race	2 (7)	NA	NA	2 (5)
Hemoglobin (mmol/l)		9.0 (7.1–10.8)	9.2 (6.7–10.1)	8.3 (8.2–8.9)	9.0 (6.7–10.8)

Median values are shown with range in brackets. Hemoglobin levels shown are at time of inclusion. *NA*, not applicable

### Parasitemia after chemoprophylaxis and sporozoite immunizations with NF135

**Fig. 2:**
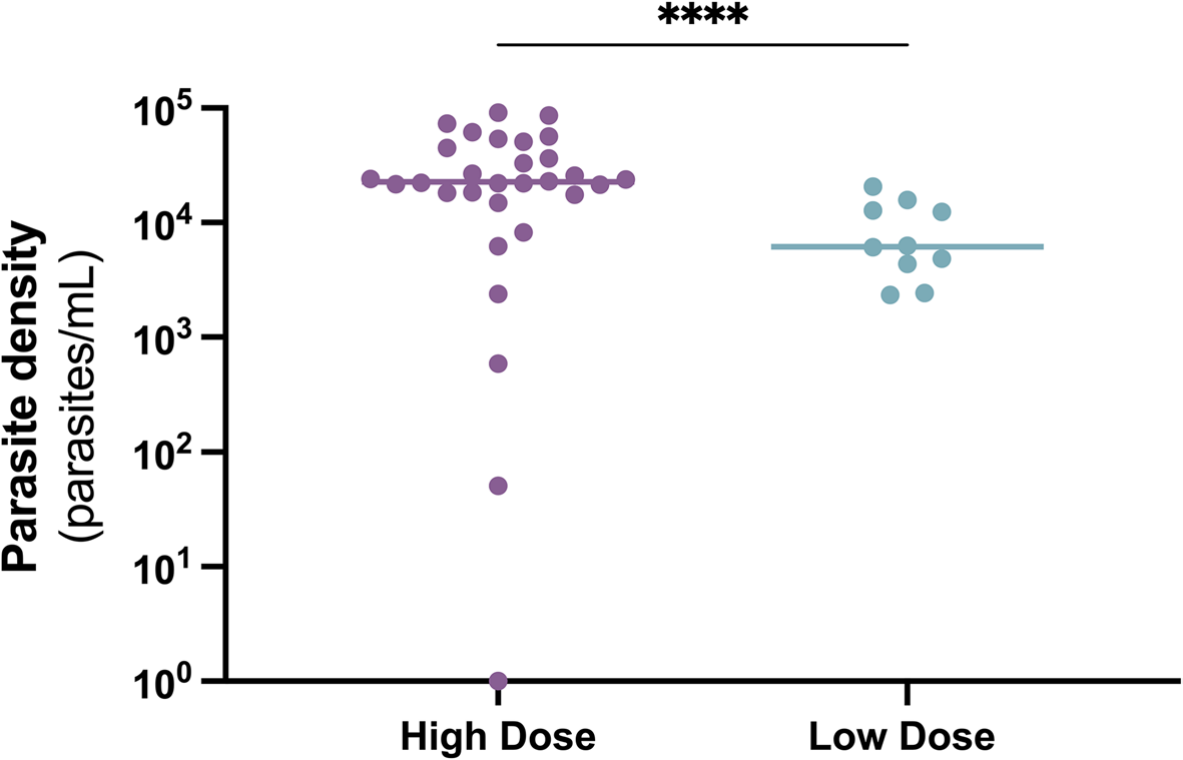
Parasitemia on day seven after immunization 1. Each dot represents one participant, the line represents the median value. Participants in the high-dose group were immunized with fifteen NF135-infected mosquitoes. Participants in the low-dose group were immunized with five NF135-infected mosquitoes. The statistical analysis performed was Welch’s *t*-test. ****p < 0001

All participants immunized with five NF135-infected mosquitoes (*n* = 10 in cohort A) developed parasitemia on day seven following their first immunization (Fig. 2, Table 2). Parasitemia was also detected in all but one of *n* = 30 high-dose participants (cohorts A and B combined) on day 7 following their first immunization. The sole cohort B participant with a negative qPCR received presumptive artemether/lumefantrine treatment that same day per protocol for this cohort, curtailing further assessments. As a correlate of liver-stage parasite biomass, the liver-to-blood inoculum, i.e., the peak of the first wave of parasitemia to emerge from the liver (on day 7) was measured [11]. The median day 7 parasitemia after the first immunization in the high-dose group was 22,614 (range 0–91,365) parasites/mL and in the low-dose group, 6181 (range 2339–20,579) parasites/mL (Table 2).

**Table 2:** Median parasitemia after immunization with NF135

	**High dose**	**Low dose**
**Infectious mosquitoes per immunization**	15	5
**Parasitemia on day 7 post-immunization**		
Immunization 1	22,614 (1–91,365)^1^	6181 (2339–20,579)^2^
Immunization 2	1 (1–1358)^3^	1 (1–1738)^4^
Immunization 3	57 (1–233,451)^3^	573 (1–8626)^4^
**Peak parasitemia following each immunization**		
Immunization 1	40,535 (1–174,050)^1^	65,550 (12,376–1,038,721)^2^
Immunization 2	1 (1–686,606)^3^	1 (1–1,670,962)^4^
Immunization 3	2536 (1–552,676)^3^	20,649 (1–5,021,767)^4^

Median parasitemia values are shown with range in brackets

^1^Cohort A + B (*n* = 30); ^2^Cohort A (*n* = 10); ^3^Cohort A only (*n* = 10); ^4^Cohort A (*n* = 9)

**Fig. 3:**
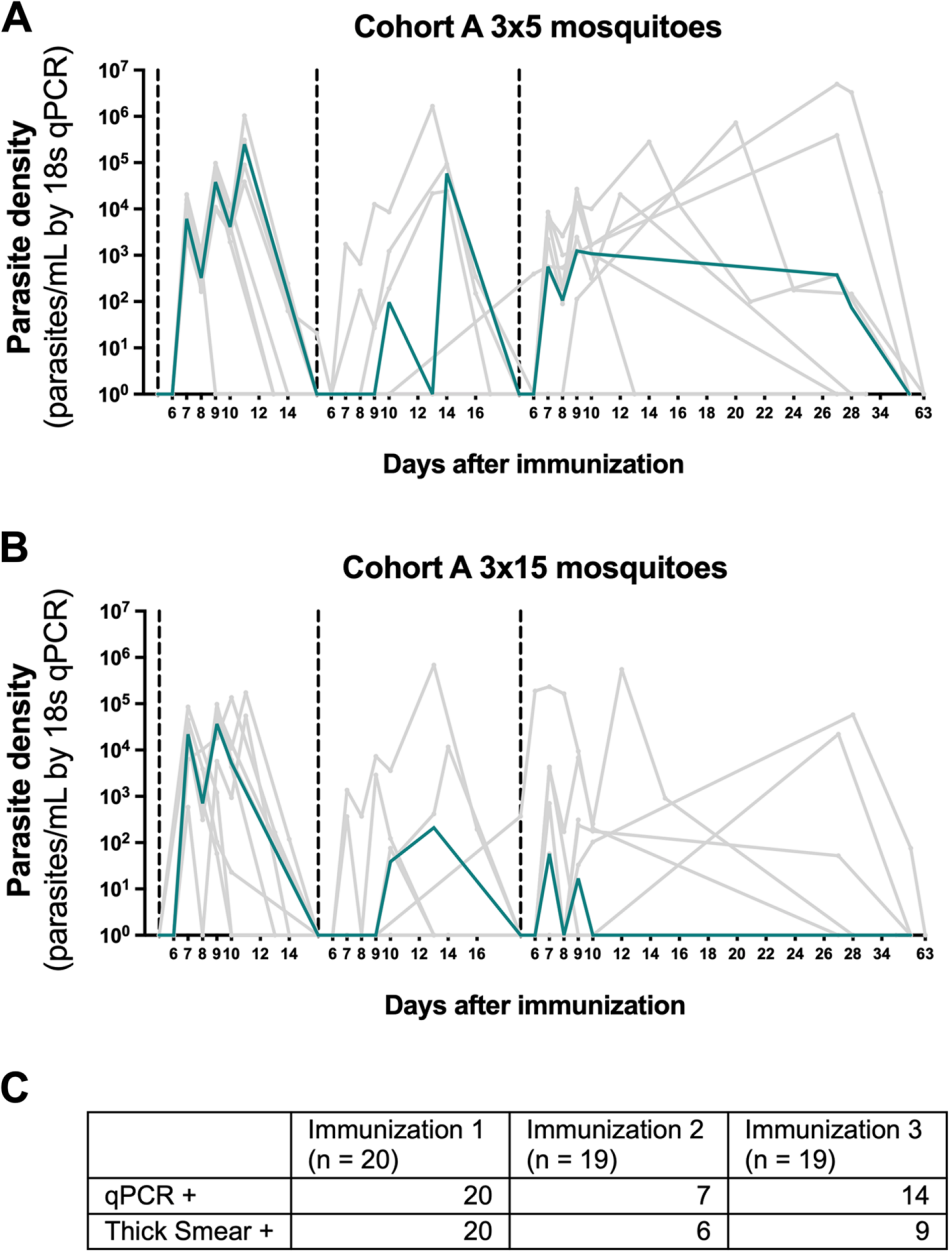
Parasitemia after immunization in cohort A. Individual parasite curves are presented in gray. The median parasite density for each group is presented in green. Dashed lines indicate an immunization. **A** Participants immunized with the low-dose of three times five NF135-infected mosquitoes. **B** Participants immunized with the high dose of three times fifteen NF135-infected mosquitoes. **C** Number of participants with a positive qPCR and/or thick smear after each immunization

Unexpectedly, all cohort A participants immunized under mefloquine prophylaxis (*n* = 20) required atovaquone/proguanil rescue treatment after the first immunization due to ongoing blood-stage multiplication. Seven participants (37%) required rescue treatment after the second immunization and fourteen (74%) participants after the third immunization (Fig. 3). Plasma mefloquine levels were sufficiently high to expect good prophylactic activity (Additional file 1: Fig S1A), and the in vitro sensitivity to mefloquine of circulating parasites had not changed compared to the parent parasite clone used for inoculation (Additional file 1: Fig. S1B). Plasma concentrations of atovaquone/proguanil were measured prior to immunizations two and three in those participants who had previously received rescue treatment and were higher than expected based on the reported half-life of atovaquone and above the predicted IC_50_ for liver-stage malaria parasites (Additional file 1: Fig. S2) [15, 19].

All twenty cohort B participants, who were treated presumptively with a standard three-day course of artemether/lumefantrine, starting on day 7, were qPCR negative by the end of treatment. Two participants nevertheless experienced a parasite recrudescence on day 19 and day 21 after immunization, respectively, despite adequate serum drug concentrations (≥ 1300 ng artemether/mL and 5200 ng lumefantrine/mL on day 3) and received successful rescue treatment with atovaquone/proguanil.

### Protection against homologous CHMI

**Fig. 4:**
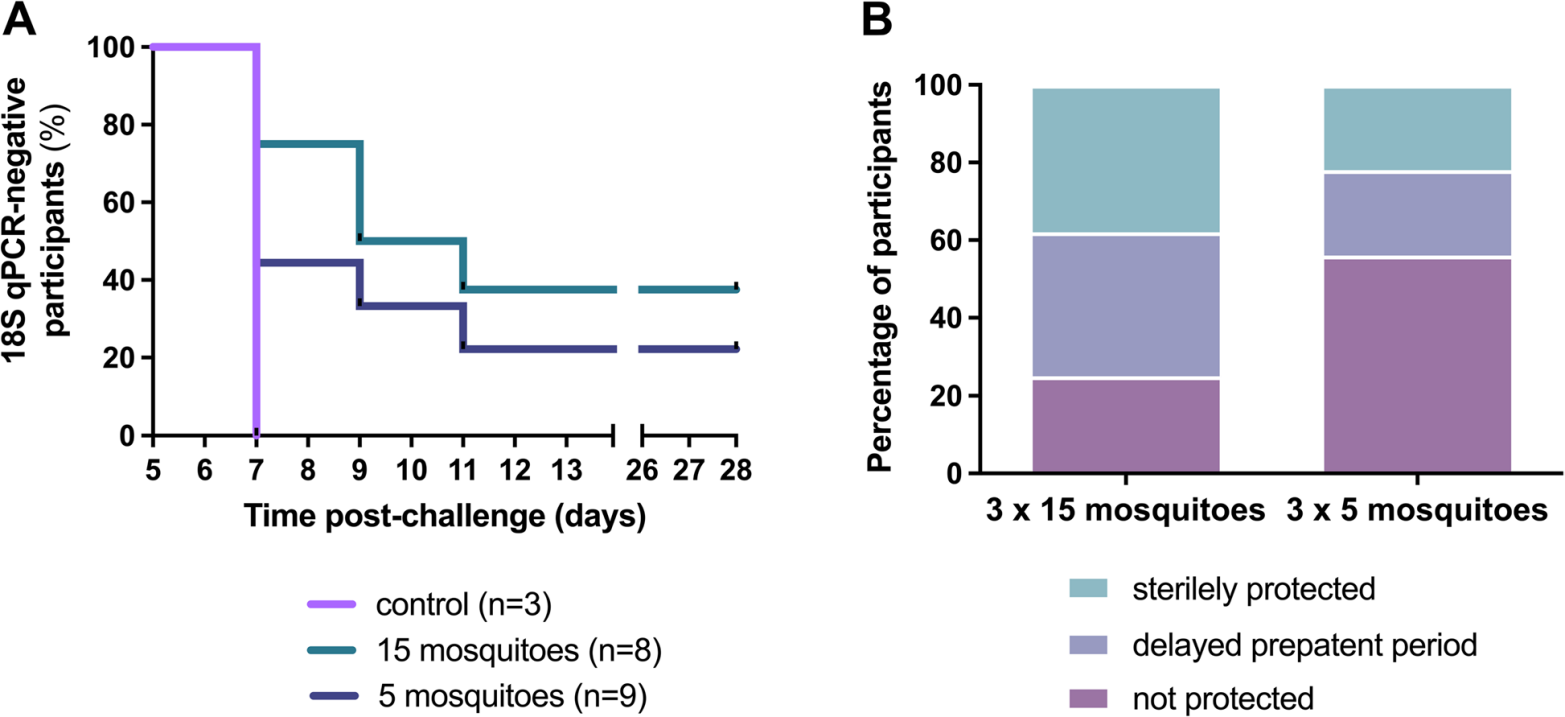
Sterile protection against *Pf*NF135 mosquito bite challenge. **A** Kaplan–Meier curve depicting percentage of participants that remained sterilely protected after homologous challenge infection with *Pf*NF135. Participants in the high-dose arm received three immunizations by bites of fifteen *Pf*NF135-infected mosquitoes. Participants in the low-dose arm received three immunizations by bites of five infected mosquitoes with the same parasite strain. The control group received no immunizations. **B** Percentage of sterilely protected partially protected and unprotected participants after controlled human malaria infection with the homologous NF135 strain in each study arm. Participants in the high-dose arm received three immunizations by bites of fifteen *P. falciparum* NF135-infected mosquitoes and participants in the low-dose arm received three immunizations by bites of five infected mosquitoes with the same parasite clone. Mosq, mosquitoes

In cohort A, nine volunteers from the low-dose group, eight volunteers from the high-dose group, and three unimmunized control participants underwent a CHMI 19 weeks after the last immunization by the bites of five NF135-infected mosquitoes. All three control participants developed a positive qPCR (> 100 parasites/mL) on day 7 (Fig. 4). Five immunized participants (29%), of whom two (22%) in the low-dose immunization group and three (38%) in the high-dose group remained qPCR negative until end of follow-up. This difference was not statistically significant between dose groups (*p* = 0.620). In total, twelve immunized participants (71%) became qPCR positive, seven (41%) on day 7, three (18%) on day 9, and two (12%) on day 11 after challenge. The median time to parasitemia was 7 days (range 7–11 days) in the low-dose group, 9 days (range 7–11) in the high-dose group, and did not differ significantly between groups (*p* = 0.36).

**Fig. 5:**
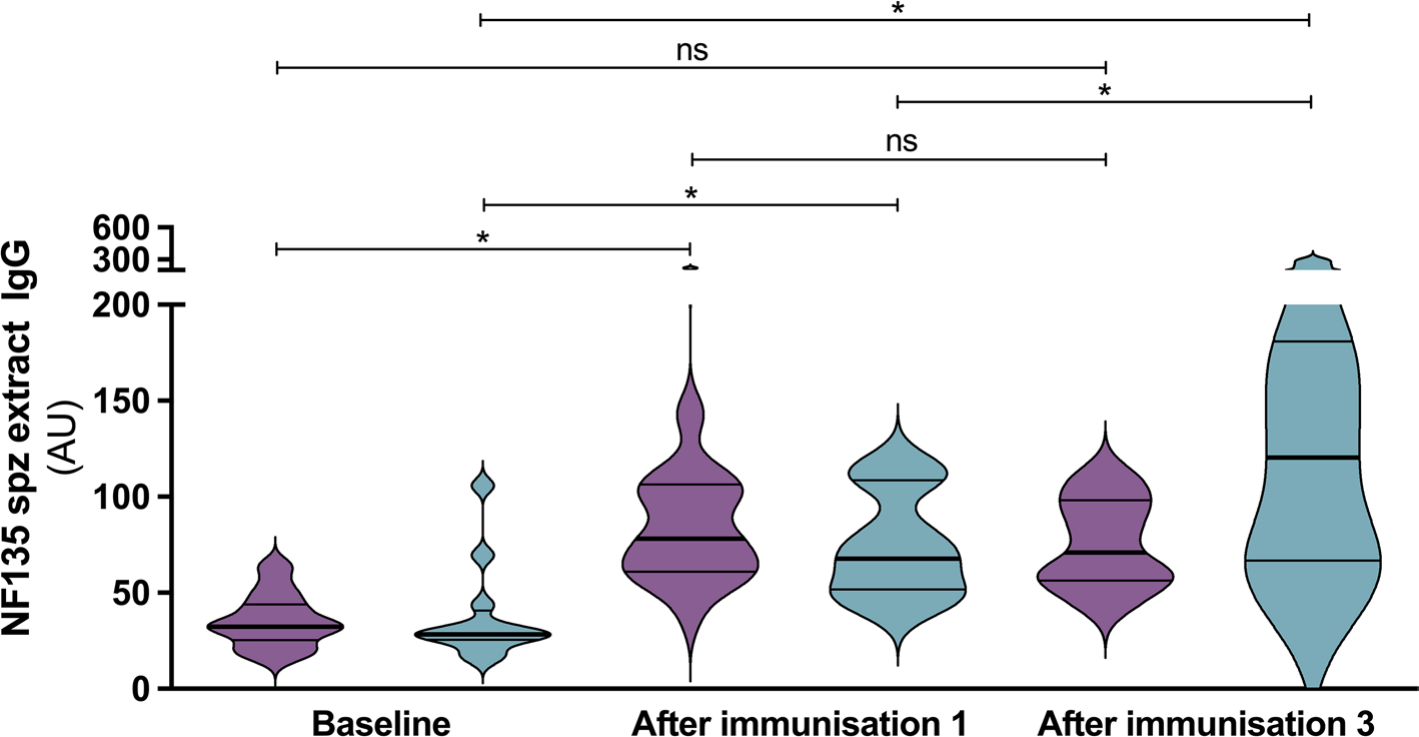
Anti-sporozoite IgG titers. IgG antibody titers against sporozoite extract in sera of cohort A participants immunized with three times fifteen *Pf*NF135-infected mosquitoes (purple, *n* = 10) and of participants in a historical comparator trial immunized with three times fifteen *Pf*NF54-infected mosquitoes (blue, *n* = 12), at baseline, after the first immunization, and after completion of the course of three immunizations. Thick and thin horizontal lines represent median and interquartile range, respectively. The Mann–Whitney *U* test was used for differences between studies. Friedman test with *post hoc* paired comparison using Bonferroni-corrected Wilcoxon signed rank test was used for comparisons of time points within studies

We determined the course of IgG antibody titers against *Pf* sporozoite extract during CPS immunizations in cohort A. Sera derived after CPS immunizations with NF54 in a historical trial served as a comparator [20]. Titers after the first immunization were similar in both trials and were significantly elevated above baseline. In contrast to the NF54 comparator trial, however, where anti-sporozoite titers against NF54, NF135, and NF175 continued to increase following immunizations 2 and 3, in cohort A participants’ sera, we observed a decreasing trend after an initial seroconversion following the first immunization (Fig. 5).

### Safety and tolerability

**Fig. 6:**
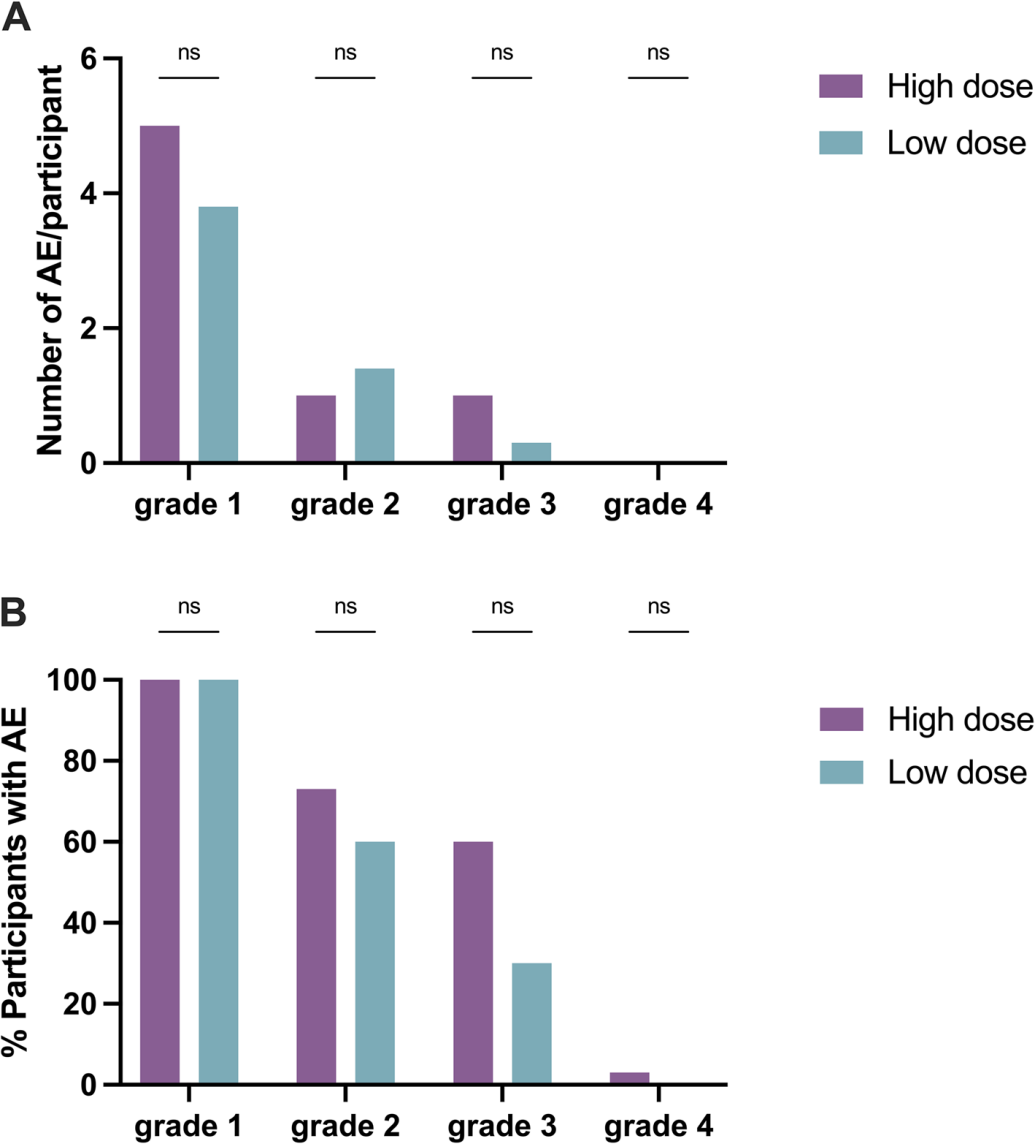
Adverse events after immunization 1. **A** Number of grade 1, 2, 3, and 4 adverse events per participant per cohort. **B** Percentage of participants who experienced a grade 1, 2, 3, or 4 adverse event per cohort. AE, Adverse event

All participants in the high- and low-dose immunization groups experienced at least one grade 1 adverse event after the first immunization. Headache and fever were the most common adverse event for participants that received a high- or low-dose immunization (Table 3). Overall, a relatively high number of grade 3 adverse events was observed: 18 out of 30 high-dose participants (60%) experienced grade 3 symptoms following the first immunization, the majority fever, as did 3 out of 10 (30%) of the low-dose participants (Fig. 6). One cohort A participant experienced mefloquine-related insomnia (D-A-S Score: 6–3-18) and was withdrawn prior to first immunization; in all other participants, mefloquine prophylaxis was well-tolerated. Two other participants registered an elevated DASS (11–6-11) and CAPE-score (> 50), respectively, both considered unrelated to mefloquine prophylaxis (Additional file 1: Table S1). Finally, one group B participant experienced acute thoracic chest pain and elevated cardiac biomarkers occurring 1 day after completion of presumptive artemether/lumefantrine treatment and in the absence of parasitemia (as evidenced by qPCR which had reverted to negative). Symptoms decreased spontaneously and the participant recovered completely. The event was reported as a serious adverse event. A succinct case description is provided in Additional file 1: Supplementary information S1. An overview of adverse events throughout the entire enrolment is shown in Additional file 1: Table S2.

**Table 3:** Systemic and local solicited adverse events throughout the study

	Low dose (*n* = 10)			High dose (*n* = 30)		
	% Of subjects	Mean number of episodes/subject	Median duration in days (range)	% Of subjects	Mean number of episodes/subject	Median duration in days (range)
**Systemic solicited**						
Headache	100	1.7	2.33 (0.17–4.79)	90	1.6	1.19 (0.01–5.00)
Fever	60	0.8	0.24 (0.02–1.46)	87	1.7	0.31 (0.02–2.93)
Nausea	80	0.8	1.70 (0.04–4.48)	43	0.6	0.58 (0.08–5.96)
Myalgia	50	0.7	2.54 (0.13–3.98)	50	0.6	1.5 (0.38–7.00)
Malaise	40	0.4	1.79 (0.65–4.46)	63	0.9	0.96 (0.08–3.96)
Fatigue	20	0.3	3 (2.79–3.21)	27	0.4	1.29 (0.15–5.21)
Chills	NA	NA	NA	30	0.3	0.15 (0.04–1.75)
Sweats	NA	NA	NA	23	0.3	0.43 (0.29–4.06)
Dizziness	20	0.3	0.04 (0.02–2.71)	23	0.2	1.02 (0.13–1.90)
Abdominal pain	NA	NA	NA	10	0.1	0.87 (0.08–1.42)
Vomiting	10	0.1	0.1	3	0.0	1.35
Diarrhea	NA	NA	NA	3	0.0	0.29
Chest pain^a^	NA	NA	NA	3	0.0	5.6
**Local solicited**^b^						
Pruritus	20	0.2	1.1 (0.86–1.33)	7	0.1	0.63

Only possibly or probably related adverse events are depicted. Adverse events of control participants and unsolicited adverse events are shown in Additional file 1: Table S2. NA: not applicable. a One participant of cohort B immunized with fifteen mosquitoes developed acute thoracic pain on day ten after immunization and was hospitalized for additional diagnostics. The serious adverse event is described in more detail in Additional file 1: Supplementary information S1. b No reports of erythema, swelling, tenderness, pain and induration.

## Discussion

Here, we conducted the first trial using the *Pf* NF135 strain in a whole sporozoite immunization regime using the CPS approach and confirmed that inoculation with NF135 results in dose-dependent liver-stage parasite burdens that are approximately twenty times higher than those previously achieved by the commonly used NF54 strain (Additional file 1: Fig. S3) [2, 20]. Immunization with NF135 under mefloquine prophylaxis resulted in dose-dependent sterile protection against a homologous CHMI challenge in a proportion of participants, and significantly delayed parasitemia in most others, almost 5 months after the final immunization.

Nevertheless, this immunization regimen was almost certainly still sub-optimal for inducing protective responses. Firstly, the unexpected bloodstage multiplication of NF135 parasites observed under mefloquine prophylaxis meant that all participants in cohort A required rescue treatment with atovaquone/proguanil following their first immunization and a proportion also following their second. Measurement of atovaquone in these participants’ blood immediately prior to their second and third immunizations revealed higher residual concentrations than anticipated based on the previously reported half-life of 2 to 3 days, and these concentrations were higher than the predicted IC_50_ for liver-stage malaria [19]. It is most likely that residual atovaquone interfered with liver-stage development during the second and third rounds of immunization in cohort A, markedly reducing parasite biomass and hence the development of protective immune responses and thwarting the interpretation of the efficacy results for this CPS regimen. A longer atovaquone half-life than described in the summary of product characteristics has been suggested previously, and this should be further evaluated as it is of relevance for both prophylactic and treatment purposes [21, 22]. Secondly, ongoing asexual parasite replication under mefloquine prophylaxis may have negatively affected the development of protective immunity by inducing immunological tolerance, as previously reported [23–25]. To assess this, we compared the development of anti-sporozoite titers, as well as protection status following challenge, between cohort A participants who either did or did not develop parasitemia following immunization 2 and following immunization 3. Although group sizes are too small to draw definitive conclusions, we found no overt differences between these four groups with regards to either anti-sporozoite titers or subsequent protection (data not shown). However, the overall trend to decreasing anti-sporozoite antibody titers following immunization 2 and 3 (Fig. 5) is supportive of the hypothesis that the immunization regimen in cohort A was compromised.

The drug susceptibility profile of NF135 differed from that based on its in vitro profile. Despite the similar mefloquine in vitro IC_50_ of NF135 and NF54, which was previously shown to be a fully effective prophylactic in NF54-inoculated volunteers, all NF135-infected participants in the current trial developed ongoing blood stage replication under mefloquine prophylaxis [26]. It has been reported that NF135 has two functional copies of the *Pf*MDR (multi-drug resistance)-1 locus [27] and *Pf*MDR has been related to mefloquine resistance in vivo [28]. This may have conferred resistance to mefloquine in vivo, but the effect may be subtle enough to go unnoticed when tested in vitro, highlighting the limitation of such assays. Secondly, two participants in cohort B experienced recrudescence following presumptive treatment with a standard three-day course of artemether/lumefantrine. Recrudescence following artemether/lumefantrine has been described previously in returning European travelers [29], mostly men, prompting some centers to recommend an alternative extended regimen, but has not previously been observed in CHMI studies in which artemether/lumefantrine was used to treat NF54 infection. *Pf*MDR has also been associated with increased artemether and lumefantrine tolerance [30–32].

Higher numbers of adverse events occurred after NF135 immunizations compared to previous NF54 CPS trials, contrary to previous observations in participants challenged with five NF135-infected mosquitoes [13]. Here, 60% of participants developed grade 3 symptoms following their first NF135 immunization compared to only 2 out of 10 (20%), 1 out of 10 (10%), and 4 out of 15 (27%) in previous trials following immunizations with the same number of NF54-infected mosquitoes [2, 26, 33]. The ongoing parasite replication despite prophylaxis may partially explain the higher number of adverse events reported in comparison to other CPS trials. Additionally, the higher liver-to-blood inocula may negatively affect the tolerability of the immunization regime by resulting in more marked malaria-associated symptoms caused by the sudden appearance and subsequent clearance under prophylaxis, or presumptive treatment, of parasite loads in excess of the thick blood smear detection limit [34]. Indeed, we observed relatively even more adverse events in cohort B participants than following the first immunization in cohort A participants receiving the same dose (not shown), possibly a result of artemether’s fast-acting parasite clearance.

A further potential safety concern is the occurrence of a cardiac serious adverse event, but it is questionable whether this can be attributed specifically to *Pf*NF135. Cardiac events have occurred previously in a small number of participants in our CHMI or CPS trials, but all previous cases involved *Pf*NF54 [35, 36]. It is noteworthy that other factors such as dose and treatment regimen have also differed between cases.

The unfavorable tolerability and drug susceptibility profile of NF135 preclude its further development as a strain for immunization under the CPS approach evaluated here. Whereas concomitant use of non-steroid anti-inflammatory drugs to decrease inflammatory responses associated with the high liver-to-blood inoculum could potentially improve tolerability [6], no practical alternative blood stage prophylactic is available. Nevertheless, neither limitation would apply when using NF135 in whole sporozoite immunization approaches that allow late liver-stage development (thus taking full advantage of its high liver-stage biomass) without resulting in the release of blood-stage parasites from the liver. Such approaches include in particular second-generation (late-arresting) genetically attenuated parasite (GAP) vaccines. An *early-arresting* liver-stage NF54 GAP-vaccine already showed excellent safety and partial induction of immunity in a recent 1-2a clinical trial [9] and a phase 1 clinical trial with a late-arresting NF54 GAP is ongoing [37] (ClinicalTrials.gov Identifier: NCT04577066).

## Conclusions

We conducted for the first time CPS immunizations with the NF135 strain of *Pf*. The CPS protocols used here with NF135 resulted in ongoing blood-stage multiplication requiring rescue treatment, thereby compromising immunization efficiency and the evaluation of optimum protective efficacy. The markedly higher liver load of NF135 compared to the commonly used NF54 strain, and its ability to induce modest protection even under suboptimal immunization conditions, support evaluation of NF135 in alternate whole sporozoite immunization approaches against malaria.

## Supplementary Information

**Additional file 1:** **Figure S1.** Mefloquine concentration and IC_50_. **Figure S2.** Atovaquone plasma concentrations. **Figure S3.** Comparison of parasite density after NF135 and NF54 immunization. **Table S1.** Registered DASS and CAPE scores in cohort A. **Table S2.** Summarized safety data per cohort. **Supplementary information S1.** Cardiac Serious Adverse Event.

## Data Availability

The datasets used and analyzed during the current study are available from the corresponding author on reasonable request.

## Abbreviations

CHMI: Controlled human malaria infection
CAPE: Community Assessment of Psychic Experiences
CPS: Chemoprophylaxis and sporozoite immunization
DASS: Depression Anxiety Stress Scales
GAP: Genetically attenuated parasites
MDR: Multi-drug resistance
*Pf*: *Plasmodium falciparum*
*Pf*SPZ-CVac: *Plasmodium falciparum* Sporozoite Chemoprophylaxis Vaccine
Radboudumc: Radboud University Medical Center

## References

[1] World Health Organization World malaria report 2021. Geneva. Licence: CC BY-NC-SA 3.0 IGO.

[2] Roestenberg M, McCall M, Hopman J, Wiersma J, Luty AJ, Gemert GJ, et al. Protection against a malaria challenge by sporozoite inoculation. N Engl J Med. 2009;361(5):468–77. 10.1056/NEJMoa0805832 19641203

[3] Roestenberg M, Teirlinck AC, McCall MB, Teelen K, Makamdop KN, Wiersma J, et al. Long-term protection against malaria after experimental sporozoite inoculation: an open-label follow-up study. Lancet. 2011;377(9779):1770–6. 10.1016/S0140-6736(11)60360-7 21514658

[4] Mordmüller B, Surat G, Lagler H, Chakravarty S, Ishizuka AS, Lalremruata A (2017). Sterile protection against human malaria by chemoattenuated PfSPZ vaccine. Nature.

[5] Sulyok Z, Fendel R, Eder B, Lorenz FR, Kc N, Karnahl M (2021). Heterologous protection against malaria by a simple chemoattenuated PfSPZ vaccine regimen in a randomized trial. Nat Commun.

[6] Mwakingwe-Omari A, Healy SA, Lane J, Cook DM, Kalhori S, Wyatt C (2021). Two chemoattenuated PfSPZ malaria vaccines induce sterile hepatic immunity. Nature.

[7] Jongo SA, Urbano V, Church LWP, Olotu A, Manock SR, Schindler T (2021). Immunogenicity and protective efficacy of radiation-attenuated and chemo-attenuated PfSPZ vaccines in Equatoguinean adults. Am J Trop Med Hyg.

[8] Seder RA, Chang LJ, Enama ME, Zephir KL, Sarwar UN, Gordon IJ, et al. Protection against malaria by intravenous immunization with a nonreplicating sporozoite vaccine. Science. 2013;341(6152):1359–65. 10.1126/science.1241800 23929949

[9] Roestenberg M, Walk J, van der Boor SC, Langenberg MCC, Hoogerwerf MA, Janse JJ, et al. A double-blind, placebo-controlled phase 1/2a trial of the genetically attenuated malaria vaccine PfSPZ-GA1. Science translational medicine. 2020;12(544):eaaz5629. 10.1126/scitranslmed.aaz5629 32434847

[10] Epstein JE, Paolino KM, Richie TL, Sedegah M, Singer A, Ruben AJ, et al. Protection against Plasmodium falciparum malaria by PfSPZ vaccine. JCI Insight. 2017;2(1):e89154. 10.1172/jci.insight.89154 PMC5214067 28097230

[11] McCall MBB, Wammes LJ, Langenberg MCC, van Gemert GJ, Walk J, Hermsen CC, et al. Infectivity of Plasmodium falciparum sporozoites determines emerging parasitemia in infected volunteers. Science translational medicine. 2017;9(395):eaag2490. 10.1126/scitranslmed.aag2490 28637923

[12] Yang ASP, van Waardenburg YM, van de Vegte-Bolmer M, van Gemert GA, Graumans W, de Wilt JHW (2021). Zonal human hepatocytes are differentially permissive to Plasmodium falciparum malaria parasites. EMBO J.

[13] Langenberg MCC, Wammes LJ,  McCall MBB, Bijker EM, van Gemert GJ, Graumans W (2018). Controlled human malaria infection with graded numbers of Plasmodium falciparum NF135.C10- or NF166.C8-infected mosquitoes. Am J Trop Med Hyg.

[14] Teirlinck AC, Roestenberg M, van de Vegte-Bolmer M, Scholzen A, Heinrichs MJ, Siebelink-Stoter R (2013). NF135.C10: a new Plasmodium falciparum clone for controlled human malaria infections. J Infect Dis.

[15] Mylan. Atovaquone/Proguanil Hydrochloride 250 mg/100 mg Film-coated tablets [updated 05 Jul 2017. Available from: https://www.medicines.org.uk/emc/product/638/smpc#gref.

[16] Ponnudurai T, Lensen AH, Gemert GJ, Bensink MP, Bolmer M, Meuwissen JH (1989). Infectivity of cultured Plasmodium falciparum gametocytes to mosquitoes. Parasitology..

[17] Hermsen CC, Telgt DS, Linders EH, Locht LA, Eling WM, Mensink EJ (2001). Detection of Plasmodium falciparum malaria parasites in vivo by real-time quantitative PCR. Mol Biochem Parasitol.

[18] Gallay J, Prod'hom S, Mercier T, Bardinet C, Spaggiari D, Pothin E (2018). LC-MS/MS method for the simultaneous analysis of seven antimalarials and two active metabolites in dried blood spots for applications in field trials: Analytical and clinical validation. J Pharm Biomed Anal..

[19] Nixon GL, Moss DM, Shone AE, Lalloo DG, Fisher N, O’Neill PM (2013). Antimalarial pharmacology and therapeutics of atovaquone. J Antimicrob Chemother.

[20] Walk J, Reuling IJ, Behet MC, Meerstein-Kessel L, Graumans W, van Gemert GJ (2017). Modest heterologous protection after Plasmodium falciparum sporozoite immunization: a double-blind randomized controlled clinical trial. BMC Med.

[21] Edstein MD, Kotecka BM, Anderson KL, Pombo DJ, Kyle DE, Rieckmann KH (2005). Lengthy antimalarial activity of atovaquone in human plasma following atovaquone-proguanil administration. Antimicrob Agents Chemother.

[22] Butcher GA, Sinden RE (2003). Persistence of atovaquone in human sera following treatment: inhibition of Plasmodium falciparum development in vivo and in vitro. Am J Trop Med Hyg.

[23] Murphy SC, Deye GA, Sim BKL, Galbiati S, Kennedy JK, Cohen KW (2021). PfSPZ-CVac efficacy against malaria increases from 0% to 75% when administered in the absence of erythrocyte stage parasitemia: A randomized, placebo-controlled trial with controlled human malaria infection. PLoS Pathog.

[24] Bejon P, Mwacharo J, Kai O, Todryk S, Keating S, Lowe B (2007). The induction and persistence of T cell IFN-gamma responses after vaccination or natural exposure is suppressed by Plasmodium falciparum. J Immunol.

[25] Ubillos I, Campo JJ, Requena P, Ome-Kaius M, Hanieh S, Rose H, et al. Chronic exposure to malaria is associated with inhibitory and activation markers on atypical memory B Cells and marginal zone-like B cells. Front Immunol. 2017;8:966-. 10.3389/fimmu.2017.00966 PMC5573441 28878766

[26] Bijker EM, Schats R, Obiero JM, Behet MC, Gemert GJ, Vegte-Bolmer M, et al. Sporozoite immunization of human volunteers under mefloquine prophylaxis is safe, immunogenic and protective: a double-blind randomized controlled clinical trial. PLoS One. 2014;9(11):e112910. 10.1371/journal.pone.0112910 PMC4232459 25396417

[27] Moser KA, Drábek EF, Dwivedi A, Stucke EM, Crabtree J, Dara A (2020). Strains used in whole organism Plasmodium falciparum vaccine trials differ in genome structure, sequence, and immunogenic potential. Genome Med.

[28] Price RN, Uhlemann AC, Brockman A, McGready R, Ashley E, Phaipun L (2004). Mefloquine resistance in Plasmodium falciparum and increased pfmdr1 gene copy number. Lancet.

[29] Sondén K, Wyss K, Jovel I, Vieira da Silva A, Pohanka A, Asghar M (2017). High rate of treatment failures in nonimmune travelers treated with artemether-lumefantrine for uncomplicated Plasmodium falciparum malaria in Sweden: retrospective comparative analysis of effectiveness and case series. Clin Infect Dis.

[30] Mwai L, Kiara SM, Abdirahman A, Pole L, Rippert A, Diriye A (2009). In vitro activities of piperaquine, lumefantrine, and dihydroartemisinin in Kenyan Plasmodium falciparum isolates and polymorphisms in pfcrt and pfmdr1. Antimicrob Agents Chemother.

[31] Ngo T, Duraisingh M, Reed M, Hipgrave D, Biggs B, Cowman AF (2003). Analysis of pfcrt, pfmdr1, dhfr, and dhps mutations and drug sensitivities in Plasmodium falciparum isolates from patients in Vietnam before and after treatment with artemisinin. Am J Trop Med Hyg.

[32] Eyase FL, Akala HM, Ingasia L,  Cheruiyot A, Omondi A, Okudo C (2013). The role of Pfmdr1 and Pfcrt in changing chloroquine, amodiaquine, mefloquine and lumefantrine susceptibility in western-Kenya P. falciparum samples during 2008–2011. PLoS One.

[33] Bijker EM, Bastiaens GJ, Teirlinck AC, Gemert GJ, Graumans W, Vegte-Bolmer M, et al. Protection against malaria after immunization by chloroquine prophylaxis and sporozoites is mediated by preerythrocytic immunity. Proc Natl Acad Sci U S A. 2013;110. 10.1073/pnas.1220360110 PMC3651438 23599283

[34] Alkema M, Yap XZ, de Jong GM, Reuling IJ, de Mast Q, van Crevel R (2022). Controlled human malaria infections by mosquito bites induce more severe clinical symptoms than asexual blood-stage challenge infections. EBioMedicine.

[35] Nieman AE, de Mast Q, Roestenberg M, Wiersma J, Pop G, Stalenhoef A (2009). Cardiac complication after experimental human malaria infection: a case report. Malar J.

[36] van Meer MP, Bastiaens GJ, Boulaksil M, de Mast Q, Gunasekera A, Hoffman SL (2014). Idiopathic acute myocarditis during treatment for controlled human malaria infection: a case report. Malar J.

[37] Franke-Fayard B, Marin-Mogollon C, Geurten FJA, Chevalley-Maurel S, Ramesar J, Kroeze H, et al. Creation and preclinical evaluation of genetically attenuated malaria parasites arresting growth late in the liver. NPJ Vaccines. 2020;7:139. 10.1038/s41541-022-00558-x PMC9636417 36333336

