# Supplementary material for: Whole sporozoite immunization with Plasmodium falciparum strain NF135 in a randomized trial
Source: BMC Med. 2023 Apr 7;21:137. doi: 10.1186/s12916-023-02788-9 (PMC10079489; doi:10.1186/s12916-023-02788-9)
Supplement: Supplementary file 1 — Additional file 1: Figure S1. Mefloquine concentration and IC50. Figure S2. Atovaquone plasma concentrations. Figure S3. Comparison of parasite density after NF135 and NF54 immunization. Table S1. Registered DASS and CAPE scores in cohort A. Table S2. Summarized safety data per cohort. Supplementary information S1. Cardiac Serious Adverse Event. [file 12916_2023_2788_MOESM1_ESM.docx]

**ADDITIONAL FILE 1**


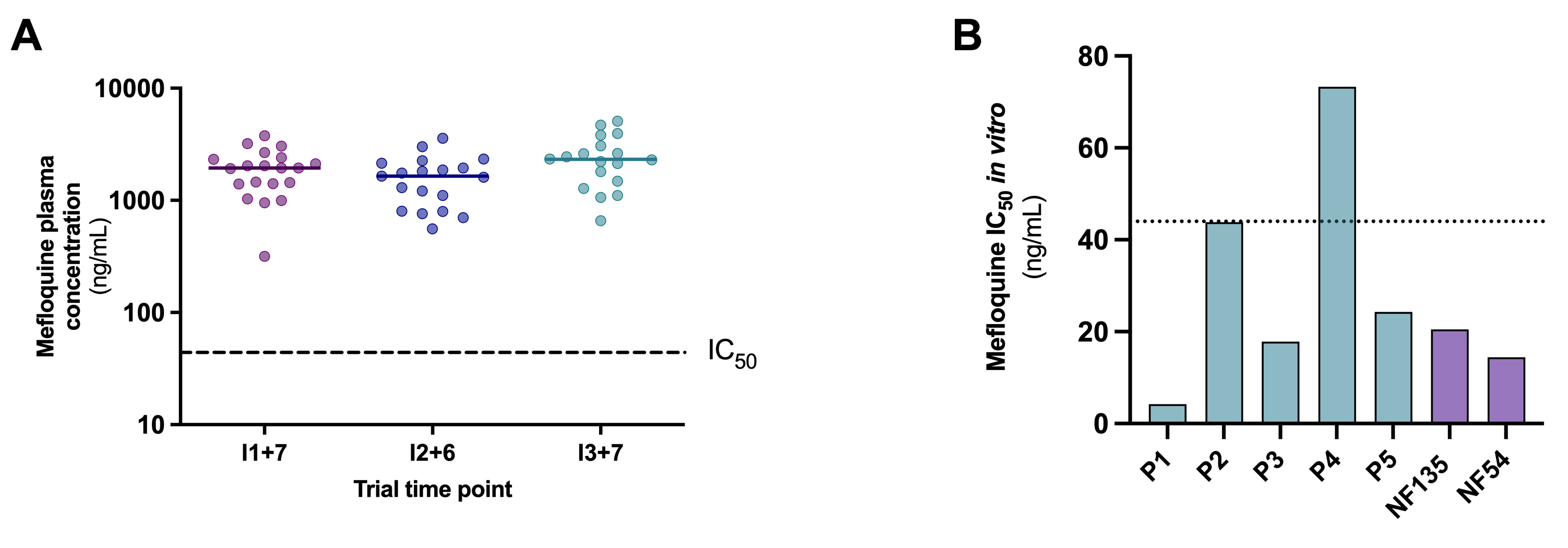


**Figure S1. Mefloquine concentration and IC_50_. (A)** Mefloquine plasma concentrations were measured in cohort A participants on day 7 after immunization 1 (I1+7), day 6 after immunization 2 (I2+6) and day 7 after immunization 3 (I3+7). Dashed line indicates the measured IC_50_ *in vitro* for mefloquine of NF135 (44 ng/mL). **(B)** The IC_50_ of NF135 parasites isolated from five participants (P1-5) was determined. Samples were collected after first detection of parasitaemia. Dashed line represents the previously measured *in vitro* IC_50_ (44 ng/mL). I: Immunization. P: participant.

**
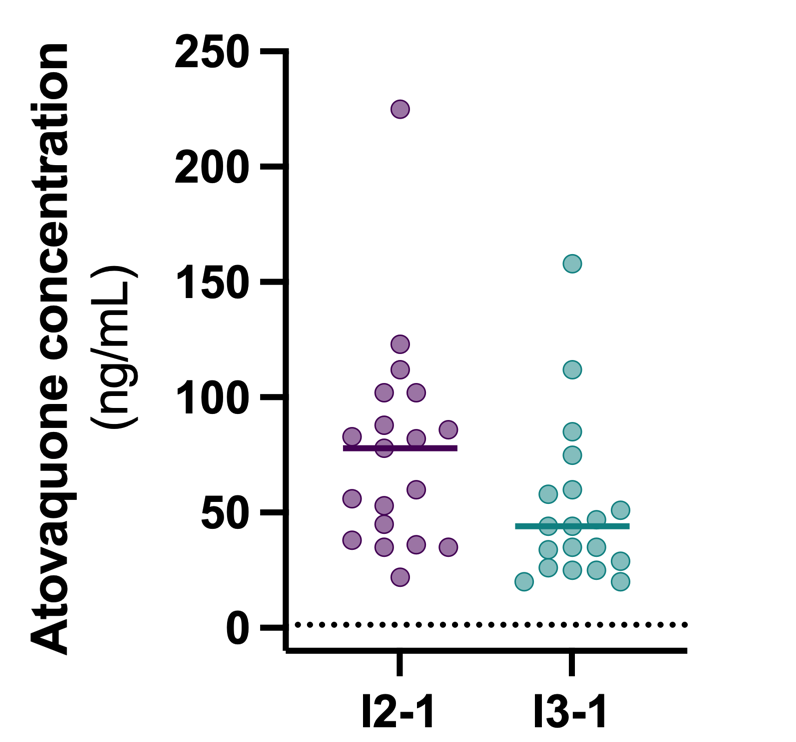
**

**Figure S2. Atovaquone plasma concentrations.** Atovaquone plasma concentration measured in participants of cohort A one day prior to immunization 2 (I2-1) and 3 (I3-1). The dashed line indicates the estimated IC_50_ *in vitro* for liver stage infection. I: immunization.


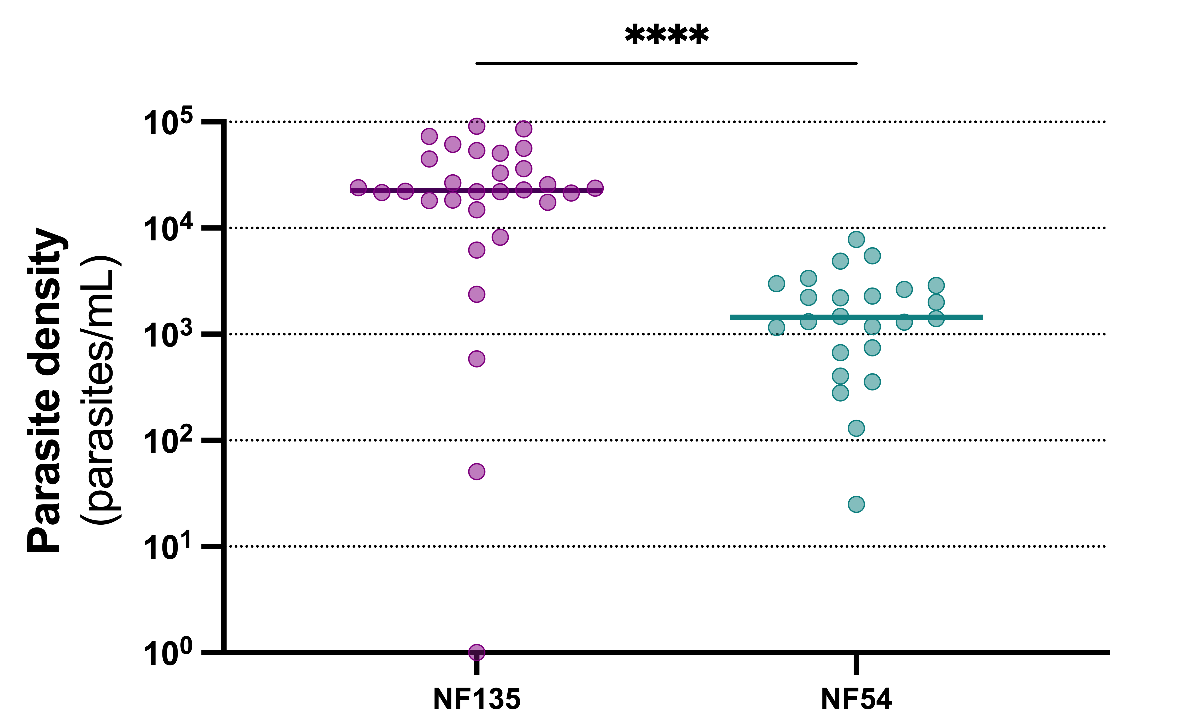


**Figure S3. Comparison of parasite density after NF135 and NF54 immunization.** Median parasitaemia on day 7 after immunization in Cohort A and B participants immunized with fifteen *Pf*NF135-infected mosquitoes, under mefloquine prophylaxis or presumptive treatment with A/L on day 7, versus participants from a previous CPS trial (BMGF2b) immunized with fifteen *Pf*NF54-infected mosquitoes under chloroquine prophylaxis. Dots represent individual parasites densities.

**Table S1. Registered DASS and CAPE scores in cohort A.**

| PARTICIPANT | DASS SCORE | | | CAPE SCORE |  |
| --- | --- | --- | --- | --- | --- |
|  | D | A | S |  |  |
|  | (0-9) | (0-7) | (0-14) |  |  |
| 1 | 5 | 3 | 9 | <50 |  |
| 2 | 7 | 3 | 0 | <50 |  |
| 3 | 1 | 3 | 1 | <50 |  |
| 4 | 0 | 0 | 0 | <50 |  |
| 5 | 3 | 0 | 2 | <50 |  |
| 6 | 0 | 2 | 0 | <50 |  |
| 7 | 0 | 1 | 6 | >50 | Score unrelated to MQ intake |
| 8 | 1 | 5 | 3 | <50 |  |
| 9 | 0 | 0 | 0 | <50 |  |
| 10 | 1 | 2 | 0 | <50 |  |
| 11 | 0 | 1 | 0 | <50 |  |
| 12 | 1 | 3 | 4 | <50 |  |
| 13 | 1 | 3 | 3 | <50 |  |
| 14 | 0 | 0 | 0 | <50 |  |
| 15 | 1 | 3 | 0 | <50 |  |
| 16 | 11 | 6 | 11 | <50 | Score unrelated to MQ intake |
| 17 | 0 | 0 | 0 | <50 |  |
| 18 | 2 | 0 | 0 | <50 |  |
| 19 | 1 | 0 | 1 | <50 |  |
| 20 | 0 | 1 | 0 | <50 |  |
| 21 | 5 | 4 | 4 | <50 | Reserve participant, excluded |
| 22 | 6 | 3 | 18 | <50 | Reserve participant. Insomnia, excluded |
| 23 | 0 | 2 | 2 | <50 | Reserve participant, excluded |

DASS and CAPE scores per participant in cohort A. In order to detect mefloquine-induced side-effects, twenty-three participants were assessed ten days after commencing prophylaxis, including three reserve participants (participant number 21-23). Two participants scored high on the DASS questionnaire: the first case (participant 16) was unrelated to mefloquine-intake, the second case (participant 22) was excluded due to insomnia. Participant 7 scored high on the CAPE questionnaire, but this was considered unrelated to mefloquine-intake. DASS: Depression Anxiety Stress Scale. CAPE: Community Assessment of Psychic Experience.

**Table S2. Summarized safety data per cohort.**

|  | Cohort A | | | Cohort A | | | Cohort B | | | Controls | | |
| --- | --- | --- | --- | --- | --- | --- | --- | --- | --- | --- | --- | --- |
|  | Immunization 3x5 (N=10) Challenge 1 x 5mosq | | | Immunization 3x15 (N=10) Challenge 1 x 5mosq | | | Immunization 1 x 15 mosq (n=20) | | | Challenge 1 x 5mosq | | |
|  | No of subjects | No of episodes | Median duration in days (Range) | No of subjects | No of episodes | Median duration in days (Range) | No of subjects | No of episodes | Median duration in days (Range) | No of subjects | No of episodes | Median duration in days (Range) |
| **SYSTEMIC SOLICITED** |  |  |  |  |  |  |  |  |  |  |  |  |
| Headache | 10 | 62 | 1,02 (0,00 - 20,13) | 10 | 35 | 0,5 (0,01 - 5,00) | 18 | 37 | 1,10 (0,01 - 4,54) | 0,27 (0,08 - 0,46) |  |  |
| Fever | 8 | 16 | 0,41 (0,02 - 1,46) | 8 | 19 | 0,58 (0,02 - 2,93) | 19 | 41 | 0,29 (0,04 - 1,88) |  |  |  |
| Nausea | 10 | 15 | 0,96 (0,02 - 4,48) | 8 | 19 | 0,63 (0,02 - 5,96) | 7 | 10 | 0,33 (0,08 - 2,54) | 2 | 3 | 1,01 (0,06 - 1,07) |
| Myalgia | 6 | 20 | 2,17 (0,08-7,39) | 6 | 11 | 1,46 (0,08 - 7,00) | 11 | 13 | 1,46 (0,38 - 4,38) | 1 | 1 | 0,75 |
| Malaise | 4 | 4 | 1,79 (0,65 - 4,46) | 7 | 13 | 0,67 (0,17 - 3,96) | 12 | 16 | 1 (0,08 - 3,00) |  |  |  |
| Fatigue | 5 | 12 | 1,75 (0,58 - 5,38) | 6 | 8 | 0,90 (0,21 - 2,96) | 7 | 10 | 1,37 (0,15 - 5,2) | 1 | 2 | 1,02 (1,00 - 1,04) |
| Chills | 2 | 3 | 0,67 (0,17 - 0,79) | NA | NA | NA | 9 | 13 | 0,15 (0,04 - 1,75) |  |  |  |
| Sweats | NA | NA | NA | 1 | 1 | 0,35 | 6 | 7 | 0,5 (0,29 - 4,06) |  |  |  |
| Dizziness | 5 | 6 | 3,21 (0,02 - 31,00) | 1 | 1 | 1 | 7,00 | 7 | 1,02 (0,13 - 1,90) |  |  |  |
| Abdominal pain | 2 | 2 | 0,16 (0,06 - 0,25) | 2 | 2 | 0,17 (0,00 - 0,33) | 3 | 4 | 0,87 (0,08 - 1,42) | 1 | 2 | 1,04 (0,54 - 1,54) |
| Vomiting | 2 | 2 | 0,01 (0,00 - 0,01) | 1 | 1 | 1,35 | NA | NA | NA |  |  |  |
| Diarrhoea | 2 | 2 | 1,40 (0,25 - 2,54) | 1 | 1 | 0,29 | NA | NA | NA |  |  |  |
| Chest pain | NA | NA | NA | NA | NA | NA | 1 | 1 | 5,6 |  |  |  |
|  |  |  |  |  |  |  |  |  |  |  |  |  |
|  |  |  |  |  |  |  |  |  |  |  |  |  |
| **LOCAL SOLICITED** |  |  |  |  |  |  |  |  |  |  |  |  |
| Pruritus | NA | NA | NA | 10 | 15 | 1,79 (0,01 - 6,91) | 2 | 2 | 0,63 |  |  |  |
| Erythema | 1 | 3 | 3,42 (2,79 - 5) | 3 | 3 | 2,46 (1,92 - 3,00) | NA | NA | NA |  |  |  |
| Swelling | 1 | 3 | 3,42 (2,79 - 5) | NA | NA | NA | NA | NA | NA |  |  |  |
| Tenderness | NA | NA | NA | NA | NA | NA | NA | NA | NA |  |  |  |
| Pain | NA | NA | NA | NA | NA | NA | NA | NA | NA |  |  |  |
| Induration | NA | NA | NA | NA | NA | NA | NA | NA | NA |  |  |  |
|  |  |  |  |  |  |  |  |  |  |  |  |  |
|  |  |  |  |  |  |  |  |  |  |  |  |  |
| **OTHER** |  |  |  |  |  |  |  |  |  |  |  |  |
| Insomnia | 4 | 7 | 0,27 (0,04 - 1,15) | 2 | 2 | 3,5 (2,00 - 5,02) | NA | NA | NA |  |  |  |
| Decreased appetite | 1 | 1 | 1 | 2 | 2 | 38,50 (1,00 - 76,00) | 6 | 6 | 2,08 (0,50 - 3,13) |  |  |  |
| Vivid dreams | 1 | 1 | 34,54 | 3 | 3 | 31,58 (8,14 - 56,10) | NA | NA | NA |  |  |  |
| Back pain | 1 | 1 | 0,15 | NA | NA | NA | 2 | 4 | 0,94 (0,38 - 1,88) |  |  |  |
| Common cold | NA | NA | NA | 2 | 2 | 0,91 (0,88 - 0,94) | NA | NA | NA |  |  |  |
| Arthralgia | 1 | 1 | 0,71 | 1 | 1 | 1,33 | 1 | 1 | 0,33 |  |  |  |
| Sadness | 1 | 1 | 12,96 | 1 | 1 | 16,54 | NA | NA | NA |  |  |  |
| Restlessness | NA | NA | NA | 1 | 1 | 16,54 | NA | NA | NA |  |  |  |
| Aften | 1 | 1 | 3,04 | NA | NA | NA | NA | NA | NA |  |  |  |
| Pyrosis | NA | NA | NA | NA | NA | NA | 1 | 1 | 0,42 |  |  |  |
| Herpes simplex | NA | NA | NA | NA | NA | NA | 1 | 1 | 10 |  |  |  |

Systemic and local solicited adverse events in cohort A, cohort B and control subjects throughout study inclusion. Participants in cohort A were immunized three times with fifteen or five mosquitoes and challenged with 5 mosquitoes. Participants in cohort B were immunized once with fifteen mosquitoes. Control participants were challenged with five mosquitoes. Only possibly or probably related adverse events are depicted. Mosq: mosquitoes. NA: not applicable.

**Supplementary information S1. Cardiac Serious Adverse Event**

A 23-year-old healthy female participant was enrolled into cohort B of the CPS135 trial in 2020. Her only known potential cardiovascular risk factor at enrolment was a self-limiting episode of tachycardia at the age of 17 of unknown cause. The participant underwent her first immunization by the bites of 15 mosquitoes infected with the NF135 strain of *Plasmodium falciparum* (*Pf*). On day 7 after the inoculation, she started presumptive treatment with artemether/lumefantrine (AL) in the morning, as per protocol, according to the standard schedule (80/480mg at t=0, t=8, t=24, t=36, t=48 and t=60 hours). At the time of the start of the AL her *Pf* parasitaemia was 72,968 parasites/mL (measured by qPCR on venous whole blood). After starting treatment with AL, she developed a fever up to 39.5°C (grade 3) the same day, with grade 1 headache, fatigue, myalgia, dizziness and nausea. In addition to AL, she received symptomatic treatment with oral or rectal paracetamol as needed up to 1000mg four times daily from day 7 through day 9 and oral domperidone as need up 10mg three times daily from day 8 through day 9. The complaints decreased in the course of day 8 after the inoculation and the next morning she was fever-free (36.5°C), with still grade 1 headache, myalgia and nausea. The qPCR on her blood was negative for *P. falciparum* from day 9 onwards. During the routine follow-up visit on the morning of day 10 after inoculation (around 08:45), she reported good recovery, no fever (35.8°C, tympanic), with only residual mild headache and myalgia. The qPCR on her blood was again negative for *Pf*. Troponin T was measured per protocol: the concentration on the morning of day 10 after the malaria infection was not elevated (5ng/L; normal value <14 ng/L).

Later that morning (10 days post-CHMI), on her way home from the routine follow-up visit, the participant developed acute chest pain over the heart. She returned to the study centre and presented with elevated troponin (max. 331 ng/L), elevated creatinine kinase (max. 230 U/L; normal value < 145 U/L) and a dynamic electrocardiogram (ECG). She was admitted to hospital for further observation, thus categorising this as a serious adverse event (SAE). She received a stat dose of 10mg metoprolol i.v. (prior to CT) and was offered nitro-glycerine sublingually as needed and oral paracetamol as needed. Her chest pain decreased spontaneously within one hour but remained present to a varying degree for three days. She was started on isosorbide mononitrate 30mg orally once daily, but this was discontinued after one day. No anticoagulation was prescribed. Troponin and creatinine kinase decreased gradually over the course of three days. Cardiac MRI, coronary computed tomography angiography (CT-A), coronary angiography (CAG) and echocardiography showed no evidence of myocarditis, Tako Tsubo or coronary occlusion / dissection. It was concluded the symptoms were probably caused by an inflammatory reaction after CHMI, without signs of myocarditis on MRI. The differential diagnosis included microvascular dysfunction. She was discharged from hospital and was followed up as an outpatient by the cardiologist over the course of a year, during which she reported sporadic episodes of chest pain during exercise. The cardiologist concluded further follow-up was not indicated after this follow-up period.
